# Clinical Lecture on Conjugal Onanism and Kindred Sins

**Published:** 1872-02

**Authors:** William Goodell

**Affiliations:** Clinical Lecturer on Diseases of Women and Children in the University of Pennsylvania


### SELECTED ARTICLES.

### CLINICAL LECTURE ON CONJUGAL ONANISM AND KINDRED SINS.

BY WILLIAM GOODELL, M.D.,
Clinical Lecturer on Diseases of Women and Children in the University of Pennsylvania.

Gentlemen,—Inasmuch as certain members of the "London Dialectical Society" have been poisoning the public mind with subtle arguments against "Over-Population and Large Families," I purpose this morning to devote my hour to some subjects which are not strictly medical, and yet with salient medical aspects—subjects in themselves vile and filthy, but which concern us as physicians. The wise son of Sirach has laid down the abstract truth, that "the knowledge of wickedness is not wisdom;" and yet, for the correct interpretation of diseases, we must intrepidly search out their causes, whether moral or physical, however loathsome or impure they may be. Receive, then, these necessary supplements to your instruction in the attitude of true students—for to such the knowledge of immorality cannot be immoral. Early in the practice of your profession, you will, I am sorry to say, find out that many of your patients, who should be the heads of large families, are practicing detestable arts to avoid offspring. You will, on the other hand, be approached—perhaps, indeed, be hard pressed—by husbands, and, for that matter, by wives also, for some method of congress unattended with the risk of impregnation. You will also be consulted for the mental and bodily infirmities resulting from these and other sexual sins. You must not, therefore, go out into the world ignorant of these evils, and consequently unable to grapple with them. It is, however, so hard a task to discuss such subjects in acceptable language, that I confess to some squeamishness, and would much rather refer you to suitable text-books. Unfortunately, although our land is flooded with a copious literature treating of the conjugal relations, with rare exceptions, it panders to our worst instincts and defiles with the slime of an impure fancy. Impudent quacks and men of battered reputation must not be your guides; far better is it for you to learn a new thrust of fence from a friendly foil, than from the stab of a foe.

My purpose is less to discuss the moral obliquity of these secret sins of the community than to show the resulting disorders. Yet I shall not limit myself to the one point of view, for the conjugal relation is two-fold in its nature: it has a moral as well as a physical expression, but so interwoven that it is hardly possible formally to dissociate them. Nor would it be wise for a physician so to do; for who, so well as he, can determine how far a disturbance in the one will affect the other? Moreover, so irreparable is the moral and physical degradation resulting from these vicious sexual relations, so damaging are they to good health and to good morals, so fatal to national prosperity, that I cannot go far astray in assaulting them with every available weapon.

You have all had a religious training, and respect the teachings of the Bible; let us see what light they throw upon the conjugal relation. The first words addressed by God to our first parents conveyed the following blessing and command: "And God blessed them, and God said unto them, Be fruitful, and multiply, and replenish the earth." The same blessing and command, in precisely the same words, were twice given to Noah. Abraham and Ishmael received the same blessing, and so did Isaac thrice in one chapter. Laban's household sent away their sister Rebekah with the same blessing. "Give me children, or else I die," was the cry of Rachel. Jacob called his offspring "the children which God hath *graciously* given thy servant;" and the same patriarch, when dying, raised himself upon his staff, in order with greater solemnity to invoke upon his beloved son Joseph "blessings of the breasts and of the womb." The Psalmist declares that "children are an heritage of the Lord: and the fruit of the womb is his reward;" and in Exodus we read that if a man "take him another wife," her food, her raiment, and her *duty of marriage*, shall he not diminish." Throughout the Old Testament you will find that fruitfulness was regarded by Jew and Gentile as the greatest of earthly blessings, and that as such it was the reward of the righteous, and as such it was withheld from the wicked. How a profanation of this blessing was regarded by God, you all know from the history of Onan, who was slain for resorting to one of the "preventive measures" in vogue at the present day.

Again, in the New Testament, we find St. Paul giving the following advice to the married Christians at Corinth: "Defraud ye not one the other, * * * that Satan tempt you not for your incontinency. Let the husband render unto the wife due benevolence; and likewise also the wife unto the husband," etc. I have not the time to quote all that the apostle says upon this subject; but, mind you, this advice was given in troublous and persecuting times—times in which the temptation was great to prevent the increase of families—times to which the words of our Saviour were especially applicable: "Woe unto them who are with child, and to them that give suck in those days."

To these Scriptural precepts and blessings you may perhaps object, that they were designed for special purposes, and that, as such, they cannot concern the present generation of men. While unwilling to admit this, I reply, that there is a natural religion as well as a revealed religion: the one, God's book; the other, Nature's—a "Second Bible," as Bacon happily terms it. You have heard what the one enjoins; now listen to the teachings of the other. Let me turn to our Case-Book and read out the history of one of our clinical patients. Some of you have seen her in my private room, but, for obvious reasons, I have not brought her before the assembled class.

A. B., aged thirty; married ten years ago; has had two children, one of them dying shortly after birth. Six years ago she and her husband came to this country and opened a small store. She was at that time in robust health, "very happy," and cheerfully waited upon their customers. For no assignable reason, her health soon began to fail, and six weeks ago she came for advice in a truly pitiable plight. To use her own language, she was "very weak and miserable;" "crying all the time;" "cannot remember anything for ten minutes;" forgets the price of the goods in her husband's store; was "constantly mislaying needful articles, and making mistakes in making change." She was "very suspicious;" fancied "that everybody was against her and talking about her," and confessed to being extremely jealous of her husband. In addition to these mental disturbances, she eructates large quantities of wind, is obstinately costive, has violent palpitations of the heart, and cannot go up one flight of stairs without getting out of breath. She often staggers, loses consciousness, and sometimes falls from vertigo; is annoyed by a persistent *globus hystericus*, and has no appetite whatever. The catamenia appear every three weeks, are abundant, but unaccompanied with pain. She has, however, a constant pain in the sacral and in the left infra-mammary region; also, a frequent desire to pass water, and much "bearing down" of all the pelvic organs.

Without wearying you with every detail, in one word, the subjective symptoms of uterine disease which she presented were more numerous and more marked than I had ever before seen in one patient. In making a vaginal examination—to which she reluctantly submitted—I was struck with the excessive sensitiveness of her tissues, and with the uncontrollable excitement under which she labored—symptoms hitherto, in my experience, limited to unmarried women addicted to self-abuse. I found the vagina crimson and hot, the womb tender to the touch, intensely congested, somewhat prolapsed, and in the first degree of retroflexion. The sound, passing through a patulous internal os, caused much pain at the fundus, and a slight hemorrhage upon its withdrawal. The os tincæ was surrounded by a collar of erosion, and plugged with the characteristic glairy secretion. Finally, she flinched from any pressure, however light, over each ovarian region. The significance of these symptoms I explained to her, but I need not to you.

She then took me aside, and, unsolicited, told me her history. Being in straitened circumstances upon their arrival in this country, and withal anxious to lay by money, she and her husband had agreed to have no more children. With this view, she had submitted to the following fraudulent and one-sided expedient: at the height of the orgasm, the husband withdraws from her person, and thus commits the crime for which Onan was punished with death. For six years such incomplete coitions had been practiced, usually as often as five times, and never less frequently than three times, a week. She had at first attributed her ill health to change of climate, but quite recently had begun to suspect its true cause from an unexpected improvement in all her symptoms during the casual absence of her husband on business.

Prompted by this suspicion, she came to consult me as to its correctness, and actually, in case it was confirmed, to learn from me some other preventive method of congress. I explained to her the sinfulness of her conduct, and urged her to receive the approaches of her husband in a normal way, as otherwise nothing could be done for her. This, however she flatly refused to do, saying she would much prefer a separation or even a divorce from him. Upon inquiry, I learned that her "husband was not the man he used to be;" that he was morose and dyspeptic, complaining much of general weakness and loss of appetite. Two weeks later, she came with much glee to say that, by a mutual agreement, this incomplete act of coition was in future to be limited to twice a week, and that she was now ready for treatment. Whereupon I refused to have anything more to do with her; and I have not seen her since.

You have heard, gentlemen, this sad history—the history of a woman whose health is shattered, whose morals are perverted, whose mind is verging toward insanity. Now, what physical law of her being, what moral obligation, has been broken? Why has Nature been so resentful, and why these fierce reprisals? These are questions which press for an answer.

The sexual instinct has been given to man for the perpetuation of his species; but, in order to refine this gift, and to set limits to its abuse, it has been wisely ordered that a purely intellectual quality—that of love—should find its most passionate expression in the gratification of this instinct. Dissociate the one from the other, and man sinks below the level of the brute. Destroy the reciprocity of the union, and marriage is no longer an equal partnership, but a sensual usurpation on the one side and a loathing submission on the other. Consider the moral effects of such shameful manœuvers: wedlock lapses into licentiousness; the wife is degraded into a mistress; love and affection change into aversion and hate. Without suffering some penalty, man cannot disturb the conditions of his well-being or trespass beyond its limitations. Let him traverse her physical laws, and Nature exacts a forfeit: dare he violate his moral obligations, an offended Deity stands ready to avenge them. That this law is immutable, witness, from the history read to you, the estrangement between husband and wife—witness his ill health and ill temper, and the wreck of body and mind to which she has been reduced.

The husband suffers mentally, because no *man* can behave in so unmanly a way without a keen sense of self-abasement—without being stung by the chastisement of remorse. Dishonor the body, the temple of the soul, and you dishonor the soul. Again, by this cowardly recoil, his enjoyment in the act is so blunted that he is tempted to seek elsewhere for those pleasures which are denied him at home. Further, he suffers physically, because, although he passes through the crisis of the sexual act, and completes it in that sense, yet, owing to his withdrawal from the person of his wife just before the moment of ejaculation, this acme of the orgasm, by the lack of the normal and necessary adjuvant—viz., the rugous and constringing vagina—is not sufficiently prolonged to wholly empty the *vasa deferentia.* Enough of the semen remains behind to tease his organs, and to kindle in him desires too importunate to tolerate any great self-control. He is thus goaded on to such sexual excesses as no brain nor brawn can long support; for a constant drain on the life-giving fluid implies a constant expenditure of nerve-force. Early exhaustion and premature decrepitude will inevitably ensue if this practice of "conjugal onanism" is persisted in.

Nor is this name a misnomer, for there is no essential difference between this habit and that of masturbation. Both injure in precisely the same way, and for precisely the same reasons. It does, indeed, seem to be the law of Nature, that man must suffer the punishment of the onanist if he parts with the "seed of another life" in any other way than in that by which it tends to become fruitful.

The wife suffers the most, because she both sins and is sinned against. She sins, because she shirks those responsibilities for which she was created; she is sinned against, because she is defrauded of her rights. Lawful congress completely performed so far satisfies an imperious instinct, that attendant local congestions are at once relieved, and to great nervous excitement succeeds a calm repose of body and mind. On the other hand, conjugal onanism provokes in her desires which keenly solicit that very gratification which is denied by the nature of the act. The excessive stimulation of the whole reproductive apparatus remains unappeased. A nervous superexcitation continues, which keeps up, as in our patient, a sexual excitement and a hyperæsthesia of the parts. By forfeiting her conjugal rights, she does not reach that timely conjuncture which loosens the tension of the coarctive muscles of her erectile tissues. Hence, the congestive orgasm of the vagina, uterus, Fallopian tubes, and of the ovaries, does not at once pass away, but persists for some time—perhaps is not wholly effaced before another incomplete coition brings a fresh installment. Thus arise engorgements, erosions, and displacements of the uterus, and inflammation of its appendages, accompanied, of course, by all those protean mental and physical manifestations which I have so often pointed out to you. She takes distorted views of life and of the marriage relation, and harbors resentment against her husband as the author of all her ills.

But we have not yet done with the train of evils. The uterine, ovarian, and vaginal plexus of veins inosculate freely with the hemorrhoidal vessels, and consequently with the *venæ portarum*. Hence, the turgescence of the one group of blood-vessels leads to engorgement of the other, and the persistent congestion of the intra-pelvic veins determines portal obstruction, and *vice versa*. The absence of valves in all these vessels, and the erectile structure of the reproductive organs, favor this turgescence. As a consequence, functional derangements of the liver are commonly associated with uterine disease. No gynæcologist has failed to observe the alternate relation of cause and effect between these two conditions. To this interdependence may we refer the obstinate costiveness, the vertigo, the loss of appetite, the dyspeptic melancholy, and the suspicious nature of our patient.

Again—for the ill effects of such practices accumulate—the very barrenness aimed at by these criminal expedients is in itself a source of disease. In sterile women, the absence of pregnancy prevents a break in the constantly-recurring catamenia, and the physiological congestion of the womb, by ceaseless repetition, is liable to become pathological. Add to this the unrelieved congestion arising from incomplete intercourse, and a prolific source of uterine and hepatic disorders is at once manifest.

I have so lately warned you against the disorders arising from excessive coitus, even when normally performed, and more especially from that indulged in during the fatigue and discomforts of the honey-moon journey, so often the starting-point of uterine disease, that it is needless for me to recur to that subject. I wish, however, in this connection, to call your attention to another source of sexual trouble, for which your advice will be sought. Either from undue ardor on the part of the husband, or from the too frigid nature of the wife, the sexual crisis with him is over before hers is reached. Such misadventures are productive not only of unhappiness, but also of disease. Here, as in conjugal onanism, the female reproductive organs are kept in a state of congestion, which is followed by like ill results, the difference being only in degree, and not in kind. For this lack of reciprocation—not, however, fatal to impregnation—you will counsel to the husband the practice of some self-denial as regards the frequency of congress, and greater self-control during the act, together with a recourse to such venal promptings as a warm and honorable affection may suggest.

But, to return from a digression, there are other artifices—nay, even equipments borrowed from the brothel—for the purpose of avoiding conception, which may well alarm publicists and statesmen; for, vile as they are, they have received the open sanction of those English political economists who forget that crime and vice and human suffering in their land are due less to "over-population and large families" than to absenteeism, to the laws of primogeniture and entail, to the grasping avarice of the rich, and to the intemperance, ignorance, and shiftlessness of the poor.* All these expedients

---

*Besides the causes here enumerated, other unsuspected correlations undoubtedly exist, for Social Science has hardly yet reached to the dignity of a science. Thus far, it consists mainly of disjointed studies and isolated observations, which yet require the *junctura callida* of collation and generalization.

operate by directly preventing the access of the spermatozoa to the uterine cavity, by destroying them, or by washing them away; but they are all hurtful equally to mind and to body. If it is hazardous for an over-heated stomach to receive a glass of iced water—its natural and accustomed beverage—how much more will it be to deluge the over-congested womb with such foreign fluids as cold or astringent injections! On the other hand, those mechanical contrivances for limiting the range of the spermatoza so blunt the pleasure as to lead to unfaithfulness or to their disuse. Moreover, in common with other teachers, I am old-fashioned enough to believe that pregnancy is a necessary condition to healthful and happy marriages; and, further, that coition is innocuous only when complete in both husband and wife, and when the germinal fluid bathes her reproductive organs. It is not always possible to trace the relation between cause and effect; some link in the chain of sequences often eludes our search. The *modus operandi* of many of our most common drugs is not known, and yet our confidence in them is not shaken, because the counterweight of our experience is greater. Therefore, for no other reason than that the common experience sanctions this postulate, I believe that the semen—aided, of course, by the general relaxation following the crisis—has a special property of allaying the congestive orgasm and the vascular turgescence of the venereal excitement.

For the limitation of families, some conscientious political economists recommend absolute abstinence. But, if the "nervous erethism" of long engagements is assigned by alienists as a common cause of insanity, and by physicians as a frequent source of uterine disturbance, what derangement of body and mind may not spring from this forced continence! Perhaps, however, we are wasting words on impossibilities. There is a widespread delusion, as old as the art of medicine itself, that intercourse after the tenth day following the cessation of the menses, is not attended with the risk of impregnation. But ovulation is not necessarily menstruation; and he who constructs domestic time-tables, or trusts to his almanac, will find that accidents may happen in the best-regulated family.

There are, in fact, no harmless or available means for thwarting Nature's plain intention; for if they should not happen to injure the body, they assuredly will the mind. How immoral must be the effect when a husband and wife meet, not "to endear each other"—as Jeremy Taylor quaintly has it—but to adjust accoutrements, to compound antidotes, and to consummate, with pre-arranged precautions and cold-blooded calculations, a union which for its perfect mental and physical fruition should be spontaneous and unrestrained! All these artifices soil the purity of thought, and degrade marriage into a carnal compact which regards alone the necessities of the flesh.

Such, then, are my views upon these so-called "misery checks" and "common-sense measures," and I feel that they cannot be gainsayed. I dare any political economist to show me one innocuous expedient whereby conception may be avoided. I challenge him to name a single preventive plan which will not do damage either to good health or to good morals. Even natural sterility is a curse. Show me a house without children, and, ten to one, you show me an abode dreary in its loneliness, disturbed by jealousy or estrangement, and distasteful from wayward caprice or unlovable eccentricity. Depend upon it, gentlemen, there are no thornless by-paths by which man can skulk from his moral and physical obligations—no safe stratagems by which he can balk God's first blessing and first command. Therefore, as hygienists, if not as moralists—as physicians, if not as patriots—as guardians of the public health, if not as philanthropists—I charge you to frown upon such practices, and take a bold stand against them. Else, see to it that in the end you are not held to a strict account for the knowledge you have this day gained.—*Philadelphia Medical Times.*

